# One-pot green synthesis of iron oxide nanoparticles from *Bauhinia tomentosa*: Characterization and application towards synthesis of 1, 3 diolein

**DOI:** 10.1038/s41598-021-87960-y

**Published:** 2021-04-21

**Authors:** Sushmitha Lakshminarayanan, M. Furhana Shereen, K. L. Niraimathi, P. Brindha, A. Arumugam

**Affiliations:** 1 Bioprocess Intensification Laboratory, Centre for Bioenergy, School of Chemical and Biotechnology, SASTRA Deemed To Be University, Thirumalaisamudram, Thanjavur, 613401 India; 2 Centre for Advanced Research in Indian System of Medicine (CARISM), SASTRA Deemed University, Thirumalaisamudram, Thanjavur, 613401 India

**Keywords:** Biomaterials, Nanobiotechnology

## Abstract

The green synthesis of NPs through plant extracts can be a modest, one-pot alternative synthesis to the conventional physical or chemical method. The prime focus of this study is to produce MNPs by the reducing effect of *Bauhinia tomentosa* leaf extract, and it was immobilized in porcine pancreatic lipase (PPL). Synthesized NPs were characterized by field emission scanning electron microscopy (FESEM), X-ray diffraction (XRD) and Raman spectroscopy, UV–Vis Spectrometry, Thermogravimetry, and Differential Scanning Calorimeter (DSC), Zeta potential test, VSM, BET and Fourier Transform Infrared Spectroscopy (FTIR). The effect of process parameters was studied, about the efficiency of immobilization are enzyme stability, the extent of enzyme reusability, its separation from products, the activity of immobilized enzyme, recovery, and its loss. Finally, the immobilized lipase was used for the synthesis of 1,3-diolein using enzyme-mediated esterification of oleic acid and glycerol. Under optimized condition (reaction temp-55 $^\circ$C; molar ratio-2.5:1; pH-7) diolein yield was achieved to be 94%. Therefore, this work was further used for the industrial production of 1,3-diacylglycerol since a perfect enzyme-catalyzed process was observed.

## Introduction

With integrated technology and science, the orientation of research from the subsisting microscopic theme towards the nanoscopic system is materializing with scientific relevance^1^. The large surface-to-volume ratio and high adsorption capacity have put nanoparticles under the good adsorbents category^2^. They are synthesized in the nanometer scale with a range of 1–100 nm^3^ and shape, size, porosity, chemical composition, etc^4^ are various factors they depend on. Medicine, electrical instrumentation, engineering, environment, buildings, biomedical and biological purposes, etc. are heterogeneous domain platforms where nanostructures have extensive applications. To date, innumerable metal and metal oxide nanoparticles are being chemically synthesized by various methods^5,6^. However, toxicity may be entangled in such methods paving the way for unsafe byproducts formation^7,8^. Therefore, for nanoparticle synthesis, a simple, environmentally friendly, and cost-effective tactic is being explored. Chief aspects that put green synthesis of NPs over chemical synthesis under the profitable category are being more economical, less labor-intensive, less toxicity, and greater stability nature^9^.

Magnetic nanoparticles that transpire to be promising practical support can trammel challenges faced by conventional NPs^10^. We can separate the magnetic NPs using a magnetic field, thereby improving their recovery, increasing the activity and stability, and also reducing steric hindrance^11^. An increase in particle stability reflects the correlation of green synthesized magnetic nanoparticles by availing the organic matter from various plant part extracts^12,13^. Also, it is a swift and reasonable method as the plant extracts containing secondary metabolites can act as both reducing and fixing agents.

Iron oxide is a transition metal oxide existing in about 16 forms, which include oxides, hydroxides, and oxide-hydroxide polymorphs, track recording unique physical and chemical properties^14,15^. This reveals the far-flung applications of iron oxide particles. Therefore, attempts for the synthesis of Iron oxide nanoparticles are in the forerun. Arularasu et al. 2018 studied the production of Fe_3_O_4_ NPs using aqueous *Kappaphycus alvarezii* (red seaweed). The degradation of textile waste by catalytic activity was effective using NPs formed by a reduction reaction and also exhibited antibacterial activity^16^. Lakshmi Pravallika et al., 2019 synthesized iron oxide nanoparticles using ethanolic extract of *Centella asiatica* (CAIONPs) by reducing ferrous and ferric chlorides which were administered to Swiss albino mice with a dosage of 2000 mg/kg body weight. Nil effects of the NPs on various tissues were revealed by histopathological studies, indicating that green synthesized NPs were safe for use in biomedical and drug delivery systems^17^. In a similar study by Izadiyan et al., 2018, iron oxide nanoparticles were synthesized using *Juglans regia* green husk extract by co-precipitation method of FeCl_3_ and FeCl_2_ and the cytotoxicity tests were performed on mouse embryonic fibroblast cell lines and human colorectal adenocarcinoma cell lines by MTT assay which had no toxic effect on both normal and cancerous cell lines^12^. Khatami et al., 2019 synthesized super-paramagnetic iron oxide nanoparticles (SPIONs) produced using a zero-calorie stevia extract which acts as both reducing and stabilizing agents. The antioxidant effect studied by DPPH assay indicated the activity of produced NPs in the acceptable range^18^. Table 1 reports the comparative studies of the synthesis of iron oxide nanoparticles from various sources reported in the literature with the present work. *Bauhinia tomentosa* is a legume species in the Fabaceae family, rich in phytochemicals such as flavonoids, quinones, tannins, etc. act as stabilizing and reducing agents in NPs production. It plays a significant role in the formation, capping, and stabilization of Iron (II) oxide nanoparticles due to the presence of phytochemical and bioactive compounds. The process was demanding due to the presence of polyphenols and antioxidants which shield the NPs from oxidation and aggregation^19^.

**Table 1:** The comparison of iron oxide nanoparticles from various sources using various methodology reported in the literature with the present work.

Source	Type of nanoparticles	Process parameters	Enzyme immobilized	Application	References
*Bauhinia tomentosa* leaves	Iron oxide	0.01 M FeCl_3_ and *Bauhinia tomentosa* leaves extract in 1:1 ratio	Porcine pancreatic lipase	Synthesis of 1, 3 diolein	Present work
Polymers, inorganic materials	Silica, Zirconia, MNPs	Use of polymers, inorganic materials	Lipases, glucosidases, cellulase	Biomaterials and biocatalysts	Sigyn Bjork Sigurdardóttiretal (2018)^55^
Chemical synthesis using AgNO_3_	Magnetic gold mesoporous silica NPs	–	Cellulase	Biofuels	Elaheh Poorakbar et al. (2018)^56^
APTES/glutaraldehyde	MNPs	–	Beta-glucosidase	Recoverable biocatalysts	Hee Joon Park et al. (2018)^57^
*Nyctanthes arbortristis* flower extract	Iron oxide	1:1 ratio of 0.2–0.5 M FeSO_4_ and *Nyctanthes arbortristis* flower extract	–	Anti-microbial	Sharma et al.^34^
*J. regia* extract	Iron oxide	1:1 ratio of *J. regia* extract and FeCl_2_ + FeCl_3_ solution	–	Cytotoxicity studies using mouse embryonic cells and human adenocarcinoma cells	Izadiyan et al.^12^
*Musa ornate* sheath extract	Iron	FeSO_4_ solution with *Musa ornate* sheath extract	–	Anti-bacterial	Saranya et al. (2017)^58^
*Langenariasiceraria*leaf extract	Iron oxide	0.01 M FeCl_3_ and *Langenariasiceraria* leaf extract in 1:1 ratio	–	Anti-microbial activity	Kanagasubbulakshmi et al. (2017)^59^
APTES/glutaraldehyde	MNPs	–	Glucose oxidase	Study of the effect of size on activity and recovery	Hee Joon Park et al. (2011)^60^
Green tea leaf extract	Iron	0.1 M FeCl_3_ and green tea leaf extract in 2:1 ratio	–	Degradation of bromothymol blue dye	George (2009)^61^

In the case of 1,3 diolein, the enzymatic approach was employed due to environmental pleasantness, safety, and mild reaction condition with improved yield. Conventionally, diacylglycerol was used to reduce the accumulation of body fat. The green synthesis of nanoparticles for enzyme immobilization has benefits to instigate the enhancement of the greater surface area, lower diffusion limitation, particle mobility, thermal stability, storage capacity, modulation of catalytic activity, cost-effective, low toxicity, effective preparation, and availability, and high productivity in terms of binding efficiency with enzymes. In the present work, to maximize the diolein yield and to improve the operational stability of the enzyme, a new synthesis was employed^20,21^. This work emphasizes on green route for the synthesis of Fe_2_O_3_ (Iron (III) oxide) nanoparticles produced from *Bauhinia tomentosa* leaf extract and to synthesize 1,3 diolein using immobilized PPL.

## Materials and methods

### Materials

The porcine pancreatic lipase (PPL) 5 (Type II, 100–500 U/mg protein using olive oil) was purchased from Sigma Aldrich Co. India. For enzyme activity analysis via the olive oil emulsion method, chemicals were obtained from Hi-Media Laboratories: dipotassium hydrogen Phosphate and Potassium dihydrogen phosphate (preparation of pH 7 buffer), gum arabic, pure olive oil, and Sodium hydroxide. Chromatographically pure monoolein and oleic acid were purchased from Sigma—Aldrich (Shanghai-china). Bradford reagent was prepared using Coomassie brilliant blue, ethanol, phosphoric acid (85% pure), and glycerol. Biosynthetic Iron (II) oxide nanoparticles were used as a support for immobilization which was prepared using leaf extract and 0.01 M FeCl_3_. The leaf from *Bauhinia tomentosa* plant was used in the present study complies with institutional, national, and international guidelines and legislation. Permission to take leaf samples were obtained.

### Biosynthesis of Fe_2_O_3_ nanoparticles

The aqueous extract of *Bauhinia tomentosa* leaves and 0.01 M FeCl_3_ solution were combined to effectuate the synthesis of iron (II) oxide nanoparticles. The extract was prepared by soaking the leaves in distilled water for 24 h^22^. The freshly prepared 0.01 M FeCl_3_ solution was added dropwise to the leaf extract in a 1:1 ratio with continuous stirring. The synthesis of nanoparticles was observed with a color change from orangish-brown solution to black precipitate. The solution was centrifuged at 4000 rpm for 15 min, followed by washing of pellet with distilled water thrice. The resultant pellet was air-dried in a hot air oven at 90ºC for 2 h to obtain black-colored, purified nanoparticles. The powder was then purified by washing with acetone^23^.

### Lipase immobilization

Porcine pancreatic lipase (PPL) was immobilized on the synthesized Fe_2_O_3_ nanoparticles separately by cross-linking. 25 mg of Fe_2_O_3_ was dispersed in 25 mL of potassium phosphate buffer to a pH of 7 in two separate flasks. Precisely weighed lipase (25 mg) from both sources was added to the above mixture separately (equal concentration of enzyme and nanoparticles: 1 mg/mL). The reaction was set at 35ºC at 150 rpm for 24 h. Filtration was employed to separate the immobilized lipase. The percentage of immobilization and specific enzyme activity was also determined. The enzyme concentration was measured by Bradford assay^24^.

### Diolein synthesis

**Figure 1:**
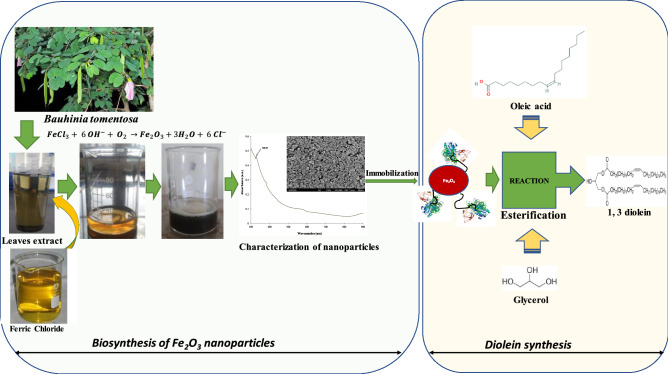
Schematic diagram of synthesis of iron oxide nanoparticles from *Bauhinia tomentosa* and 1, 3 diolein production.

The enzymatic esterification of oleic acid and glycerol was done with the support of immobilized PPL. The reaction was carried out in a 50 mL flask on a rotary shaker at 200 rpm^25^. To make up the reaction mixture, 1.5 mmol of oleic acid, 0.5 mmol glycerol, 10 mL of t-butanol, and an appropriate amount of immobilized lipase was added (15% (wt%) of the substrate). 4 Å molecular sieves were added into the reaction mixture to remove the water content. 50 µL of the sample was taken out from the reaction mixture and centrifuged to obtain the supernatant and analyzed by HPLC^26^. All the experiments were done in triplicates. Overall process layout for synthesis of iron oxide nanoparticles from Bauhinia tomentosa and 1, 3 diolein production was presented in the Fig. 1.

### Analysis of the samples

According to^20,27^, external standards of 1-monoolein, 2-monoolein, 1,2-diolein, 1,3-diolein, and triolein were used to prepare 8 different concentrations of calibration solution. The results were examined by Shimadzu 20A HPLC along with an evaporative light scattering detector (ELSD). 2µL of sample and 1 mL of acetone was entirely mixed, out of which 20µL of the sample was injected in a chromatographic column—C18 column (5 µm, 250 mm × 4.6 mm) (Dikma technology, PLATISIL ODS, china). To analyze the reaction mixture, gradient elution with acetonitrile and dichloromethane was used under various reaction conditions mentioned (100/0,0–4 min; 90/10,12–25 min; 70/30, 25–30 min; 20/80, 35–45 min; 100/0, 55–60 min). The flow rate was maintained at 1.5 mL min$^{ - 1}$, Column temperature at −40 °C, drift pipe temperature at −70 °C, and nitrogen pressure was set at 320kpa. The reaction times of 2-monoolein, 1-monoolein, 1,3-diolein, 1,2-diolein and triolein were 3.753, 4.534, 23.128, 23.883 and 42.925 min respectively.

## Results and discussion

Iron (II) oxide nanoparticles were synthesized using *Bauhinia tomentosa* leaf extract. Transformation in color was observed from an orangish-brown solution to a black precipitate. The nanoparticles were washed with water and acetone thrice and dried at 90 ºC in a hot air oven to achieve black-colored purified nanoparticles.

Ferric Chloride solution of 0.01 M concentration gets reduced to Ferric oxide and gets precipitated in the leaf extract. This reaction materializes in the company of oxidizing agents like Vitamin E^28^. Phytochemicals such as flavonoids, quinines, tannins, etc. act as stabilizing agents in nanoparticle production in the presence of a polar solvent, water. Phenols and terpenoids may play a significant role in the formation, capping, and stabilization of Iron (II) oxide nanoparticles^29^. Also, due to Surface Plasmon Resonance, a color change was observed. For measuring adsorption of material onto planar metal or the surface of metal NPs many standard tools are formed based on SPR^30^.

### Characterization of Fe_2_O_3_ nanoparticles

#### UV–vis spectrometry

**Figure 2:**
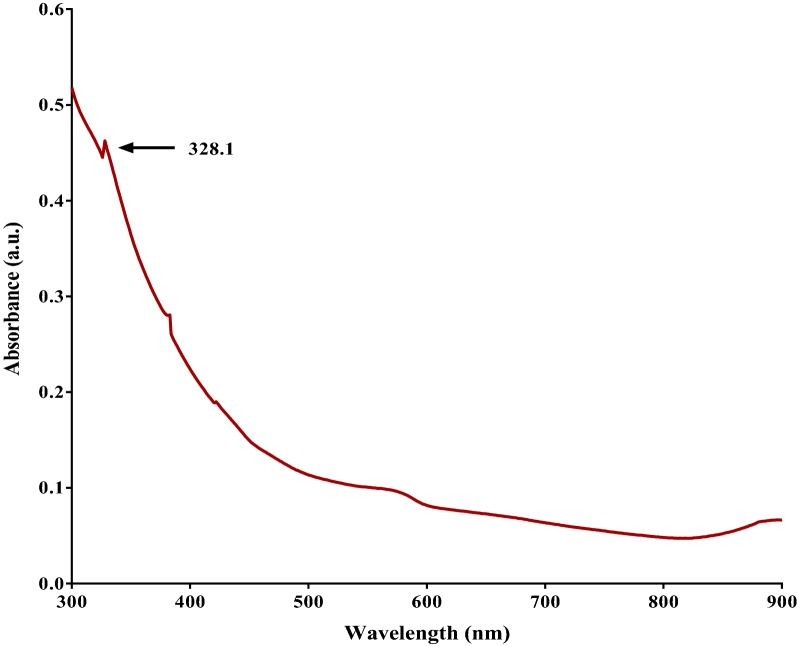
UV-V is Spectroscopy of Fe_2_O_3_ Nanoparticles synthesized from *Bauhinia tomentosa* leaf extract using the FeCl_3_ solution. The characteristic peak formed at 328 nm shows the formation of nanoparticles.

UV–Vis Spectrometry has revealed the characteristic formation of nanoparticles during color change based on the absorption spectra. A scanning wavelength measurement from 300 to 900 nm was executed to reveal a peak value at 328 nm which indicated the formation of nanoparticles (Fig. 2). A characteristic peak at 328 nm confirmed the formation of Fe_2_O_3_ Nanoparticles^31^.

#### Fourier transform infrared spectroscopy

**Figure 3:**
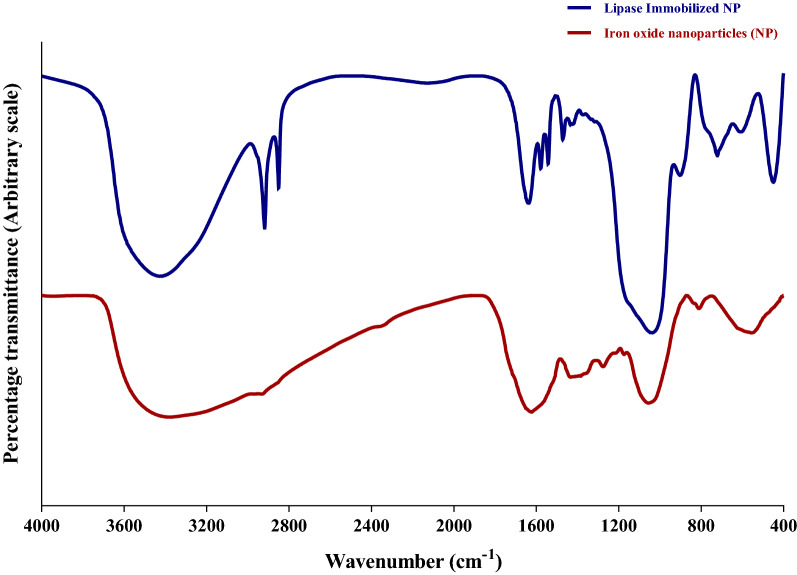
FT-IR Spectrum of bare Fe_2_O_3_ Nanoparticles and lipase immobilized nanoparticles.

FTIR is ascribed to functional groups (=C–H, C=O, N–O, C–O, C–N) present in the compound (Fig. 3). FTIR spectroscopic studies confirm the presence of amides, phenols, nitrogen, and aromatic compounds that has a strong binding affinity with Fe and thus play a significant role in reducing and capping ferrous ions^32^. The spectrum reveals characteristic peaks at 3385.9 cm^−1^ stretching to O–H, 1624.7 cm^−1^ stretching to N=O, 1172.4 cm^−1^and 1055.6 cm^−1^ stretching to O–C, 810.8 cm^−1^ and 555.7 cm^−1^ stretching to Fe–O stretches of Fe_2_O_3_^7^. The synthesis of Fe_2_O_3_ nanoparticles extracted from *Bauhinia tomentosa* aqueous leaf extract has been evinced by these chemical groups.

The stretching of carbonyl groups in lipase was observed by a broadening of peaks in the range of 3345 cm^−1^–3650 cm^−1^ for both forms of the immobilized formulation. The amplitude of peaks at 3483, 2922, 1652, and 650 cm^−1^ increased dramatically, suggesting that lipase was effectively immobilized^33^. The peak strength of covalently immobilized lipase, on the other hand, decreased (Figure. 3), indicating that the enzyme-nano relationship was stable. Because of the pairing of NH-bending with CN stretching, the band based at 1541 cm^−1^ was credited to the amide II of enzymes.

#### Thermogravimetry and differential scanning calorimeter

**Figure 4:**
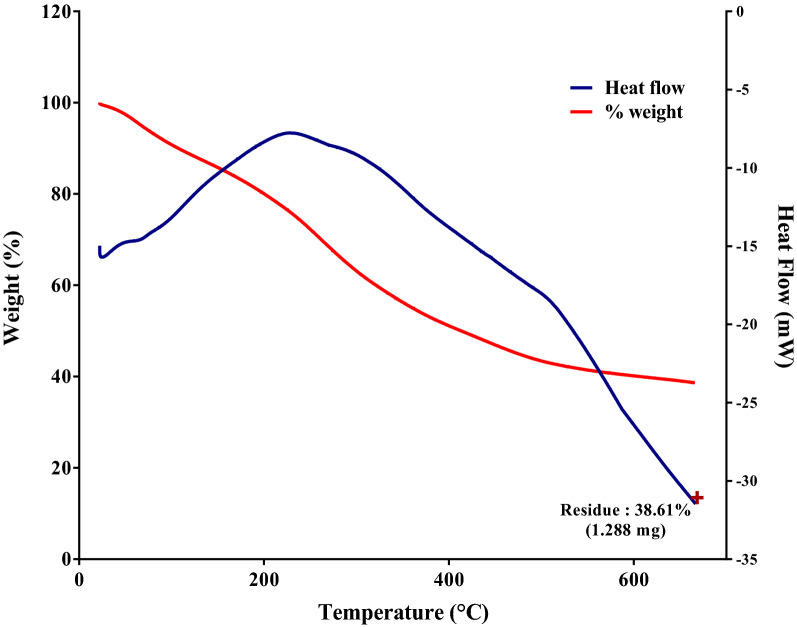
DSC-TGA for Fe_2_O_3_ nanoparticles synthesized from *Bauhinia tomentosa* leaf extract using FeCl_3_ solution.

Mass changes of a sample as a function of temperature in scanning mode are examined by TGA (dynamic TGA) (Fig. 4). The physical and chemical properties of materials, as a function of increasing temperature, can be determined. This decomposition/degradation temperature bear witness to mass changes in the materials. The approximate temperature of Fe_2_O_3_s transition of interest was found to be around 930 $^\circ$C. Characterization of coatings on NPs by evolved gas analysis can be achieved using TG-DSC techniques. DSC was grounded on the differences in the amount of heat required to increase the temperature of the sample. In combination with TGA, it was applied to study melting point, gas transitions, and exothermic decompositions. The graph depicts that the decomposition melting of the sample starts at around 250 $^\circ$C and ends at about 700 $^\circ$C revealing that the sample was Iron (II) oxide^34^. At a temperature of around 180 °C, the TGA curve showed a weight loss of around 3.0446 percent in the study. This weight loss may be attributed to the removal of water molecules removed by nanoparticles from the atmosphere, during which the sample weight is almost stable, indicating the sample's thermal stability.

#### Zeta potential and field emission-scanning electron microscopy

**Figure 5:**
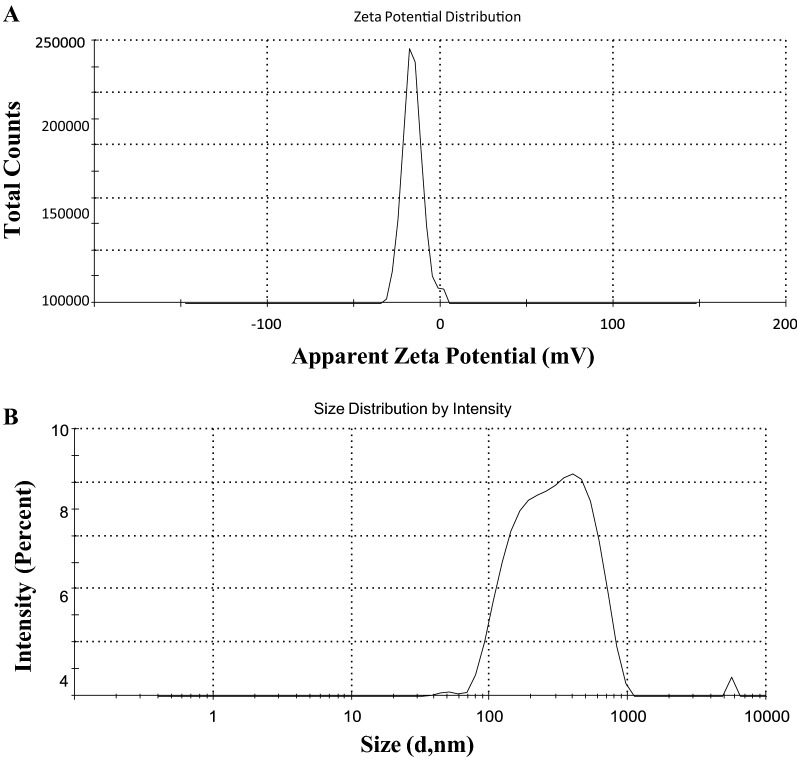
(**A**) Zeta Potential for Fe_2_O_3_synthesized nanoparticles. From the graphical result, the potential was found to be −16 mV which was a good indication for the formation of nanoparticles. (**B**) Zeta sizer for Fe_2_O_3_synthesized nanoparticles from *Bauhinia tomentosa* leaf extract.

**Figure 6:**
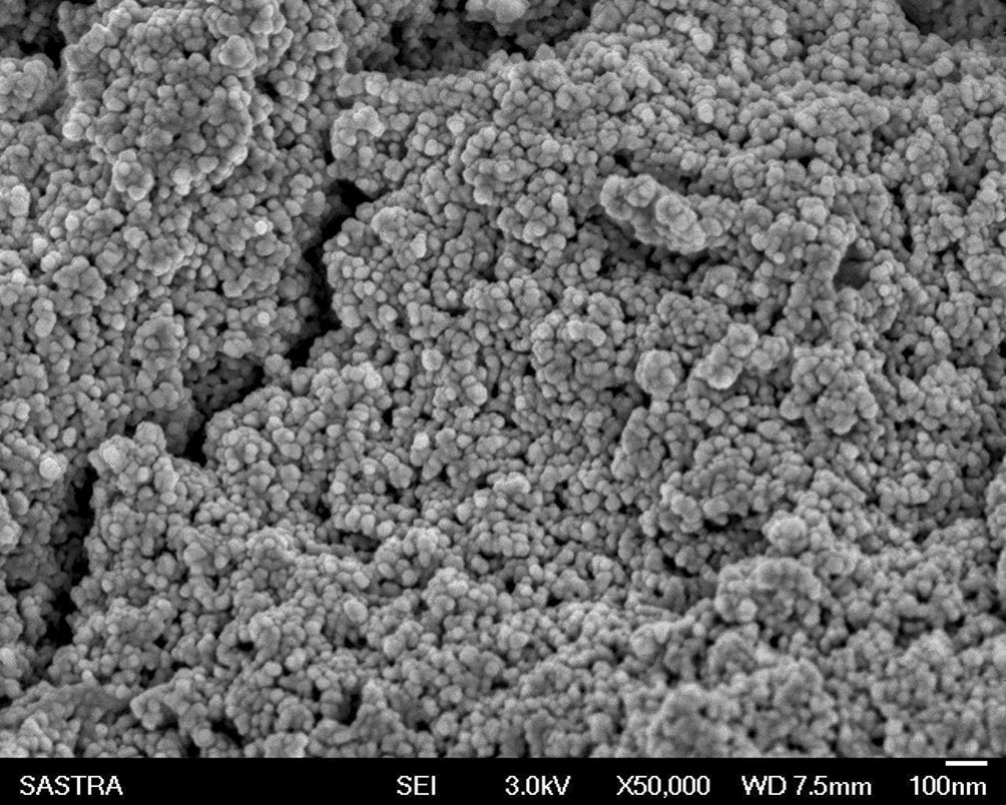
Scanning electron microscope (SEM) image of synthesized Fe_2_O_3_ nanoparticle.

Size is an important factor to define NPs although considerable debate exists on the size threshold to distinguish NPs from bulk materials. The particles were dispersed in water with a dielectric constant of 78.5, a refractive index of 1.33, and a viscosity of 0.887 cP^35^. A potential of −16 mV was found which was a good manifestation for nanoparticle formation. The potential difference between the EDL (electric double layer) of electrophoretically mobile particles and the layer of dispersant around them at the slipping plane is reflected by the zeta potential (Fig. 5A). It is also termed electrokinetic potential, the potential at the slipping/shear plane of a colloid particle moving under the electric field. Therefore, the particle size distribution and magnitude of electric charge at the particle surface are determined^36^. Also, a zeta sizer was employed to determine the size of the particles. The size distribution was scanned by intensity (Fig. 5B). However, due to differences in dispersion co-efficient and cluster formation, it did not provide accurate results. The FE-SEM image revealed the size of the synthesized nanoparticles (Fig. 6). Thus, eminently meticulous results were provided by FE-SEM. The average size was observed to be around 70 nm which is acceptable.

#### X-ray diffraction

**Figure 7:**
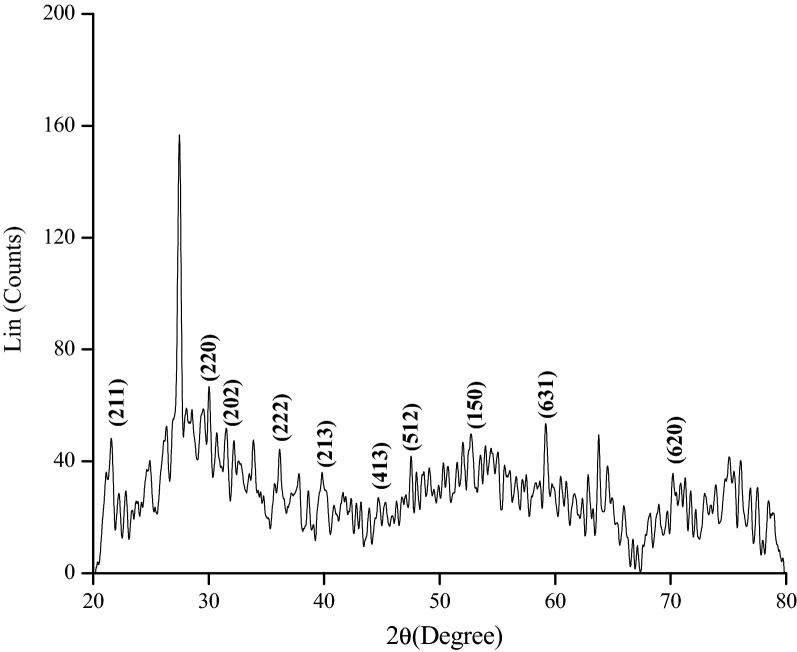
X-ray diffraction (XRD) pattern for synthesized iron oxide (Fe_2_O_3_) nanoparticle. The figure illustrated that the peak intensity was found to be highest at a theta scale value of 27.4.

X-Ray Diffraction (XRD) was performed to understand the crystalline structure of the nanoparticles. The sample consisting of fine grains of crystalline material to be studied was usually in powdered form^37^ (Fig. 7). At a theta scale value of 27.4, the peak intensity was found to be the highest. The intensity count and percent intensity were found to be 169 and 100%, respectively. The JCPDS file 019–0629 closely matched with the XRD pattern observed in this study showing the characteristic peaks at 2θ of 21.6, 25.77, 31.06, 40.68, 45.45, 53.49, 56.44, and 61.11 corresponding to the face-centered cubic phase of (211), (220), (202), (213), (431), (512), (150) and (613) planes, respectively. The presence of strong and sharp peaks of Fe_2_O_3_ crystals is attributed to the highly crystalline nature. The characteristic peaks at 2θ of 70.91 correspond to the crystal planes of (620) of crystalline Fe_3_O_4_-NPs, respectively. Material match analysis revealed the presence of Fe_2_O_3_ at higher amounts in the sample with trace amounts of Fe_3_O_4_. This indicated the formation of Iron (II) oxide.

#### Brunauer–Emmett–Teller (BET) surface area analysis

**Figure 8:**
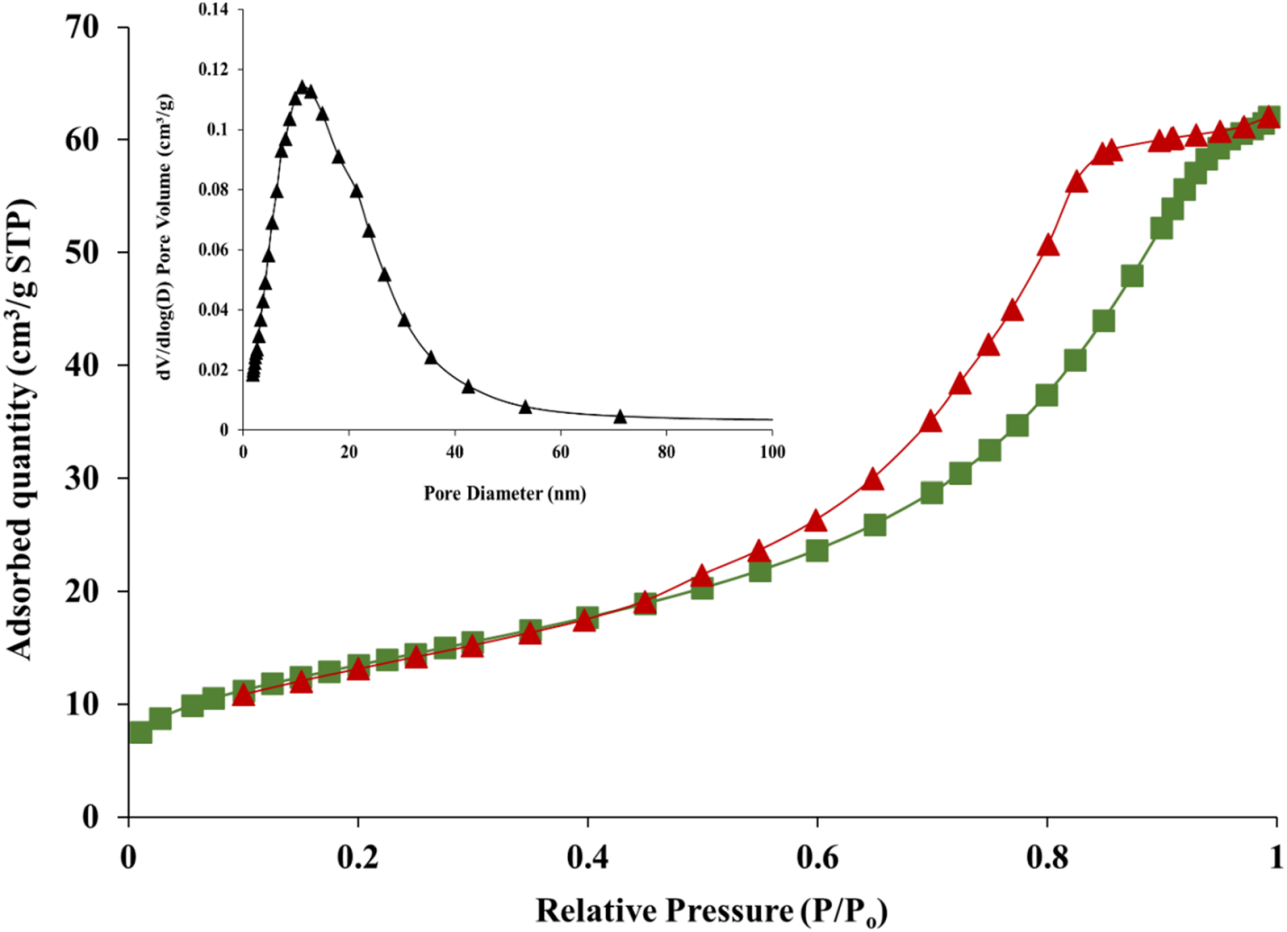
N_2_ adsorption–desorption graph with a variation of pore diameter with respect to dV/dlog(D).

N_2_ adsorption/desorption isotherms at liquid nitrogen temperature were used to determine the precise surface area (Brunauer–Emmett–Teller, BET) pore size and pore volume of the samples. Figure 8 displays the outcomes of the BET analysis^38^. The synthesized iron oxide nanoparticles display TYPE IV adsorption–desorption isotherm. The prepared nanoparticles showed Brunauer–Emmett–Teller (BET) surface area, pore-volume, and diameter were calculated to be 48.8 m^2^/g with 0.096 cm^3^/g and 7.9 nm respectively. From the adsorption–desorption isotherm, it can be noticed that around 62.04 cm^3^/g of nitrogen was adsorbed at maximum relative pressure (P/P_0_) of 1^39^. The hysteresis pattern shows that the condensation occurred approximately from 0.4 to 0.9 (P/P_0_) (Fig. 8). These findings suggest that these particles have a large surface area and are nanometer in size. In contrast to the other samples, the iron oxide Np sample had the highest surface area and had a very small particle size along with a strong adsorption property, according to the BET report^40^.

#### Vibrating sample magnetometer (VSM) analysis

**Figure 9:**
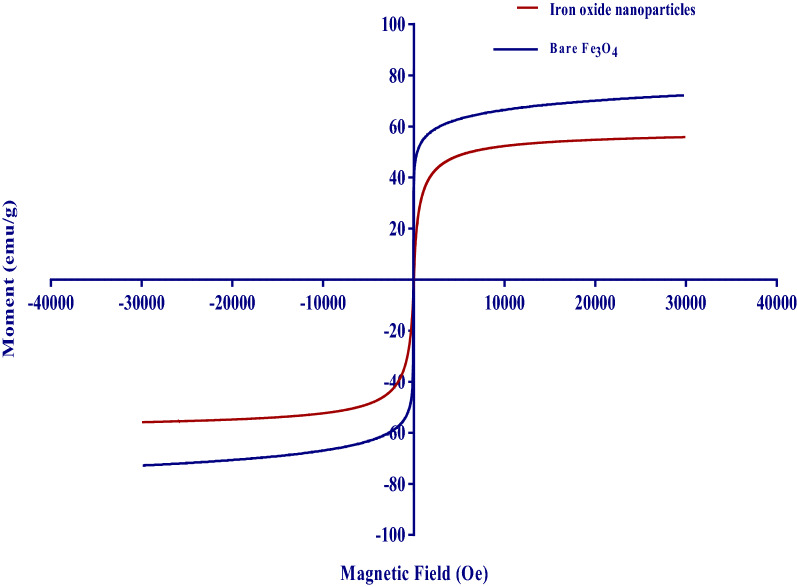
VSM curves for the prepared iron oxide nanoparticle and bare Fe_3_O_4_.

A vibrating sample magnetometer was used to test the magnetic properties of the iron oxide nanoparticles, at room temperature, the hysteresis loops of the bare Fe_3_O_4_ and iron coated NPs are shown in Fig. 9^41^. As the magnetic field is withdrawn from both prepared NPs, the magnetization decreases from a plateau state to zero. This action clearly shows superparamagnetic behavior^42^. The bare Fe_3_O_4_ and nanoparticles have a saturation magnetization (Ms) of 87.8 emu/g and coercivity (Ce) of 4.09 Oe, suggesting that they have strong magnetic properties. Similarly, iron-oxide nanoparticles show (Ms) of 55.83 emu/g and (Ce) of 1.02 Oe. It can also be categorized as a soft magnet material category due to its low coercivity value. These findings indicate that our synthesized nanoparticles exhibit a suitable behavior and can be used for enzyme immobilization and ease of recovery after the completion of the reaction.

### Determination of enzyme activity

The percentage immobilization of PPL on iron-oxide nanoparticles was found to be 70.1%. The enzyme activity of PPL covalently immobilized on the Fe_2_O_3_ matrix was calculated to be 266 U/mL^43^. Either by covalent bonding or adsorption, the interaction of enzymes with the NPs surface provides the inkling of the operational stability of enzymes^24^. However, a conclusion has been derived by the higher enzyme activity of PPL immobilized on Fe_2_O_3_ nanoparticles that this matrix could be more competitive compared to other matrices. The catalyst turnover number (TON) and the turnover frequency (TOF) for the immobilized enzyme on iron (II) oxide nanoparticles for the synthesis of 1, 3 diolein are 1.17 mol/g and 0.0039 mol/g.min.

### Effect of various reaction parameters

**Figure 10:**
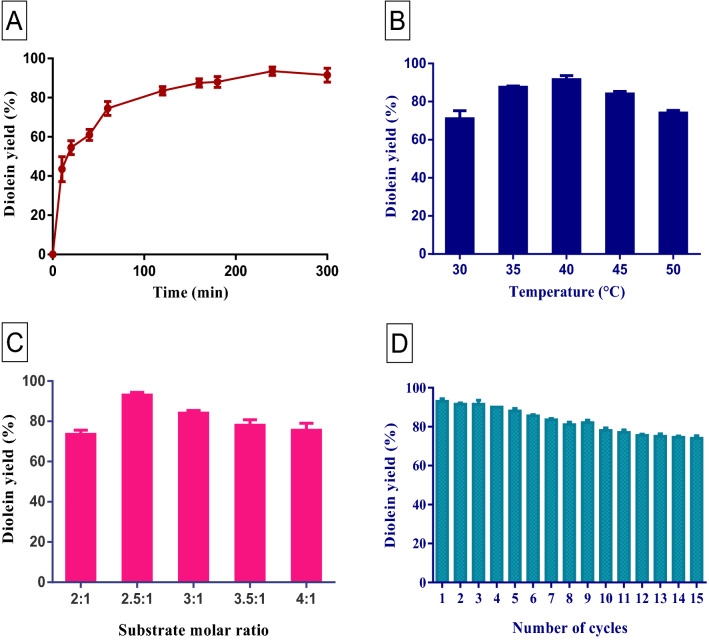
Effect of process parameters for the conversion of 1,3-diolein synthesis via esterification of oleic acid with monoolein catalyzed by immobilized *Porcine pancreatic* lipase. (**A**) The time course percentage conversion. (**B**) Temperature. (**C**) Substrate molar ratio. (**D**) Reusability studies.

Finding the effect of various parameters that affect the diolein yield based on reaction time, temperature, substrate molar ratio, and reusability of the immobilized enzyme has been pivoted in this study (Fig. 10). An indispensable role is played by the reaction temperature in biocatalysts. Higher temperature results in the deactivation of the enzyme. This work entails five different temperatures (40, 45, 50, 55, 60, and 65 °C) and was ascertained to observe the diolein yield. At 55 $^\circ$C, diolein yield reaches the highest value of 92.5%. More than that range, the yield and initial reaction rate of diolein get decreased and simultaneously acyl migration will take place which results in triolein formation and diolein yield reaches optimum value after 7 h of reaction time.

To investigate the optimum level of the substrate molar ratio based on the yield of 1,3 diolein, different ranges were taken to experiment (2:1, 2.5:1, 3:1, 3.5:1, and 4:1). The diolein yield will not be tremendously affected by an escalation in the molar ratio of oleic acid to glycerol. But higher concentrations of oleic acid will simultaneously diminish the yield of 1,3 diolein formation. Therefore, based on molar ratios, no significant difference was observed in the diolein yield. And from this work, it was observed that the substrate molar ratio of oleic acid to glycerol (2.5:1) shows the highest yield of diolein as 94%. Cost efficiency is imperatively influenced by the reusability of the immobilized enzyme^44^. The operational stability of immobilized lipase was carried out under optimized conditions. From the results, it was observed that 90% of the original activity was maintained until 10 cycles and in this case, a maximum yield of 1,3 diolein was achieved^45^. Therefore, the catalytic activity of the enzyme was not lost, and also it was proved how effectively the enzyme binds to the matrix. From the above results, it was clearly shown that Fe_2_O_3_ nanoparticles were an eminent matrix for lipase (PPL) immobilization. Therefore, the immobilization of enzymes on a solid support such as nanoparticles is more advantageous due to improved stability, enhanced thermal efficiency and pH, increased enzyme loading, and reusability with simple handling and separation making the process feasible with maximal yield. Table 2 represents the detailed comparison studies reported in the literature for the synthesis of 1,3 diolein using lipase catalysis with the present work. It was found that a higher yield of 1,3 diolein was obtained with the lipase immobilization on the iron oxide nanoparticles and also the immobilized enzyme eases the process of recovery and reuse. This reduces the overall production cost of the 1,3 diolein synthesis.

**Table 2:** The comparison of 1,3-diolein synthesis using free/immobilized lipase catalytic process reported in the literature with the present work.

Process parameters	Enzyme immobilized	1,3-diolein yield (%)	References
Temp-45 $^\circ$C, 200 rpm, molar ratio of oleic acid / monoolein of 1.5, solvent weight of 80% (oleic acid), and water activity of 0.33	Lipozyme TL IM	–	Dai et al.^46^
Ratio of 1,3-diolein to 1,2-diolein -7:1, Specific activity of enzyme- 34.5U/g, Incubation temp-50 $^\circ$C, Flow rate-1.5 ml/min	Lipase	86	Bi et al.^47^
Final concentration of 1,3-DAG- > 76%, Molar ratio oleic acid to glycerol- 2.8:1	*Rhizopus oryzae* lipase	> 76	Zhao et al.^48^
Temp-40 $^\circ$C, 12 h, Centrifuge-5000 rpm, Diethyl ether/ Hexane ratio-1:1v/v	Immobilized lipase from *Penicillium Expansum*	87.3	Duan et al.^49^
Temp- 45 $^\circ$C, 200 rpm, Molar ratio of oleic acid and glycerol-1:0.4	Lipozyme TL IM	61.1	Wang et al.^50^
Temp- 30 $^\circ$C-35 $^\circ$C, Duration-8 h	Novozyme 435, Lipozyme RM IM	90.4	Wang et al.^21^
Temp- 60 $^\circ$C, Molar ratio of oleic acid to monoleic-1.2:1	Novozyme 435	93.7	Duan et al.^20^
Temp-62.4 $^\circ$C, Molar ratio of oleic acid to glycerol-2.4 and 4.8	Novozyme 435	87.8	Duan et al.^27^
Water activity-0.53, Column temp-40 $^\circ$C, Drift pipe Temp-70 $^\circ$C, Nitrogen pressure-320 KPa	Novozyme 435	24.1	Duan et al.^52^
Temp-60 $^\circ$C, 200 rpm	Novozyme 435	81.4	Duan et al.^53^
Melting point- < 45 $^\circ$C, Pressure-3 mmHg Vaccum	*Rhizomucor miehei* lipase	84.6	Rosu et al.^54^
Nano particle synthesis: 0.01 M FeCl_3_ and *Bauhinia tomentosa* leaves extract in 1:1 ratio 1,3 diolein synthesis: Reaction temperature-55 $^\circ$C; molar ratio-2.5:1; pH-7	*Porcine pancreatic* lipase immobilized on iron oxide nano particles	94	Present work

## Conclusion

This work highlighted the green synthesis of Fe_2_O_3_ nanoparticles from *Bauhinia tomentosa* leaf extract and it was efficaciously implemented for lipase immobilization. Moreover, it was the pragmatic approach for enhancing the synthesis of 1,3-diolein by the esterification of oleic acid and glycerol. The phenolic compounds present in *Bauhinia* leaf extract play a vital role in boosting up the stability of Fe_2_O_3_ nanoparticles. The distinct characteristics, size, and shape of Fe_2_O_3_ nanoparticles were identified using FTIR and SEM analysis. XRD, TGA, and UV–Vis spectroscopic techniques were used to recognize the crystallographic structure, thermal stability, and optical behavior of the green synthesized nanoparticles were studied. Further, due to the high stability, effectiveness, enzyme activity, greater safety, low energy consumption, and high product quality of the immobilized lipase, it was employed for 1,3-diolein synthesis which will gain momentum for various applications. Finally, this greener optimistic work will aid in the large-scale synthesis of 1,3 diolein using the effective binding of immobilized lipase.

## Data Availability

The datasets used during the current study are available from the corresponding author on reasonable request.
