# Author Correction: One-pot green synthesis of iron oxide nanoparticles from *Bauhinia tomentosa*: Characterization and application towards synthesis of 1, 3 diolein

**DOI:** 10.1038/s41598-021-97276-6

**Published:** 2021-08-31

**Authors:** Sushmitha Lakshminarayanan, M. Furhana Shereen, K. L. Niraimathi, P. Brindha, A. Arumugam

**Affiliations:** 1 Bioprocess Intensification Laboratory, Centre for Bioenergy, School of Chemical and Biotechnology, SASTRA Deemed To Be University, Thirumalaisamudram, Thanjavur, 613401 India; 2 Centre for Advanced Research in Indian System of Medicine (CARISM), SASTRA Deemed University, Thirumalaisamudram, Thanjavur, 613401 India

Correction to: *Scientific Reports* 10.1038/s41598-021-87960-y, published online 21 April 2021

The original version of this Article contained an error in the spelling of the author Sushmitha Lakshminarayanan which was incorrectly given as Sushmitha Lakshmnarayanan.

The original Article has been corrected.

